# Supplementary material for: HIVprotI: an integrated web based platform for prediction and design of HIV proteins inhibitors
Source: J Cheminform. 2018 Mar 9;10:12. doi: 10.1186/s13321-018-0266-y (PMC5845081; doi:10.1186/s13321-018-0266-y)
Supplement: Supplementary file 2 — Additional file 2. Source code of HIVProtI web server. [file 13321_2018_266_MOESM2_ESM.zip › HIVprotI_Source-code/hivprotI/weka-3-6-6/doc/weka/gui/visualize/package-summary.html]

weka.gui.visualize


---


|  |  |  |
| --- | --- | --- |
| **Overview** \| **Package** \| Class \| **Tree** \| **Deprecated** \| **Index** \| **Help** \| **Weka's home** | |  |
| **PREV PACKAGE**   **NEXT PACKAGE** | **FRAMES**    **NO FRAMES**     **All Classes** |  |


---

## Package weka.gui.visualize

| **Interface Summary** | |
| --- | --- |
| **AttributePanelListener** | Interface for classes that want to listen for Attribute selection changes in the attribute panel |
| **Plot2DCompanion** | Interface for classes that need to draw to the Plot2D panel \*before\* Plot2D renders anything (eg. |
| **PrintableHandler** | This interface is for all JComponent classes that provide the ability to print itself to a file. |
| **VisualizePanelListener** | Interface implemented by a class that is interested in receiving submited shapes from a visualize panel. |

| **Class Summary** | |
| --- | --- |
| **AttributePanel** | This panel displays one dimensional views of the attributes in a dataset. |
| **AttributePanelEvent** | Class encapsulating a change in the AttributePanel's selected x and y attributes. |
| **BMPWriter** | This class takes any JComponent and outputs it to a BMP-file. |
| **ClassPanel** | This panel displays coloured labels for nominal attributes and a spectrum for numeric attributes. |
| **JComponentWriter** | This class takes any JComponent and outputs it to a file. |
| **JPEGWriter** | This class takes any JComponent and outputs it to a JPEG-file. |
| **LegendPanel** | This panel displays legends for a list of plots. |
| **MatrixPanel** | This panel displays a plot matrix of the user selected attributes of a given data set. |
| **Messages** | Messages. |
| **Plot2D** | This class plots datasets in two dimensions. |
| **PlotData2D** | This class is a container for plottable data. |
| **PNGWriter** | This class takes any JComponent and outputs it to a PNG-file. |
| **PostscriptGraphics** | The PostscriptGraphics class extends the Graphics2D class to produce an encapsulated postscript file rather than on-screen display. |
| **PostscriptWriter** | This class takes any Component and outputs it to a Postscript file. |
| **PrintableComponent** | This class extends the component which is handed over in the constructor by a print dialog. |
| **PrintablePanel** | This Panel enables the user to print the panel to various file formats. |
| **ThresholdVisualizePanel** | This panel is a VisualizePanel, with the added ablility to display the area under the ROC curve if an ROC curve is chosen. |
| **VisualizePanel** | This panel allows the user to visualize a dataset (and if provided) a classifier's/clusterer's predictions in two dimensions. |
| **VisualizePanelEvent** | This event Is fired to a listeners 'userDataEvent' function when The user on the VisualizePanel clicks submit. |
| **VisualizeUtils** | This class contains utility routines for visualization |

---


|  |  |  |
| --- | --- | --- |
| **Overview** \| **Package** \| Class \| **Tree** \| **Deprecated** \| **Index** \| **Help** \| **Weka's home** | |  |
| **PREV PACKAGE**   **NEXT PACKAGE** | **FRAMES**    **NO FRAMES**     **All Classes** |  |


---
